# Low-Density Polyethylene Film Biodegradation Potential by Fungal Species from Thailand

**DOI:** 10.3390/jof7080594

**Published:** 2021-07-23

**Authors:** Sarunpron Khruengsai, Teerapong Sripahco, Patcharee Pripdeevech

**Affiliations:** 1 School of Science, Mae Fah Luang University, Chiang Rai 57100, Thailand; 6171102001@lamduan.mfu.ac.th (S.K.); 6351102001@lamduan.mfu.ac.th (T.S.); 2 Center of Chemical Innovation for Sustainability (CIS), Mae Fah Luang University, Chiang Rai 57100, Thailand

**Keywords:** biodegradation, LDPE film, fungi, Fourier transform infrared spectroscopy (FTIR), scanning electron microscopy (SEM), gas chromatography-mass spectrometry (GC-MS)

## Abstract

Accumulated plastic waste in the environment is a serious problem that poses an ecological threat. Plastic waste has been reduced by initiating and applying different alternative methods from several perspectives, including fungal treatment. Biodegradation of 30 fungi from Thailand were screened in mineral salt medium agar containing low-density polyethylene (LDPE) films. *Diaporthe italiana*, *Thyrostroma jaczewskii*, *Collectotrichum fructicola*, and *Stagonosporopsis citrulli* were found to grow significantly by culturing with LDPE film as the only sole carbon source compared to those obtained from *Aspergillus niger*. These fungi were further cultured in mineral salt medium broth containing LDPE film as the sole carbon source for 90 days. The biodegradation ability of these fungi was evaluated from the amount of CO_2_ and enzyme production. Different amounts of CO_2_ were released from *D. italiana*, *T. jaczewskii*, *C. fructicola*, *S. citrulli*, and *A. niger* culturing with LDPE film, ranging from 0.45 to 1.45, 0.36 to 1.22, 0.45 to 1.45, 0.33 to 1.26, and 0.37 to 1.27 g/L, respectively. These fungi were able to secrete a large amount of laccase enzyme compared to manganese peroxidase, and lignin peroxidase enzymes detected under the same conditions. The degradation of LDPE films by culturing with these fungi was further determined. LDPE films cultured with *D. italiana*, *T. jaczewskii*, *C. fructicola*, *S. citrulli*, and *A. niger* showed weight loss of 43.90%, 46.34%, 48.78%, 45.12%, and 28.78%, respectively. The tensile strength of LDPE films cultured with *D. italiana*, *T. jaczewskii*, *C. fructicola*, *S. citrulli*, and *A. niger* also reduced significantly by 1.56, 1.78, 0.43, 1.86, and 3.34 MPa, respectively. The results from Fourier transform infrared spectroscopy (FTIR) reveal an increasing carbonyl index in LDPE films culturing with these fungi, especially *C. fructicola.* Analysis of LDPE films using scanning electron microscopy (SEM) confirmed the biodegradation by the presence of morphological changes such as cracks, scions, and holes on the surface of the film. The volatile organic compounds (VOCs) emitted from LDPE films cultured with these fungi were analyzed by gas chromatography-mass spectrometry (GC-MS). VOCs such as 1,3-dimethoxy-benzene, 1,3-dimethoxy-5-(1-methylethyl)-benzene, and 1,1-dimethoxy-decane were detected among these fungi. Overall, these fungi have the ability to break down and consume the LDPE film. The fungus *C. fructicola* is a promising resource for the biodegradation of LDPE which may be further applied in plastic degradation systems based on fungi.

## 1. Introduction

A trillion tons of plastic materials are produced around the world each year at a rate of 12% [1,2]. Thailand is one of the major countries that produces plastic materials in Southeast Asia, with a total production of around 27.8 million tons in the year 2020 [3]. LDPE is a long chain polyethylene and is known as one of the most widely used plastic materials worldwide [4,5]. It has been widely produced in the form of plastic bags for packaging materials due to its effectiveness and versatile properties, such as being cheap, durable, light weight, energy efficient, and easy to produce [6,7]. Plastic bags are evaluated as one of the major plastic wastes causing global environmental pollution due to their lightness, high tensile strength, and resistance to water and microbial attacks [8,9]. These plastic bags are generally released in the environment by dumping into landfills, rivers, and oceans [10]. In dumping areas, terrestrial animals usually consume plastic bag wastes along with foodstuffs, which disrupts their digestive systems, resulting in the deaths of millions of animals [9,11,12].

Accumulated plastic bag wastes are generally degraded by physical, chemical, and photo-degradation processes [2,4]. However, these processes are very expensive, and release pollutant organic compounds and toxic irritant products into the environment, such as furans and dioxins [4]. The toxic compounds result in soil infertility, preventing degradation of normal substances, and depletion of underground water sources [13,14]. Recently, biodegradation by microorganisms has been reported to degrade plastic bag wastes through anaerobic processes in soil and composts, producing carbon dioxide, water, and methane [15,16]. Therefore, biodegradation is considered as an eco-friendly process for the removal of plastic bags which may be applied as an alternative method for plastic waste management.

Biodegradation is known as the decomposition of plastics by using microbial activity involving four steps: biodeterioration, depolymerization, assimilation, and mineralization [17,18]. The biodegradation rate of microorganisms is dependent on various factors, such as substrates, environment, temperature, and the molecular weight of the plastic material [12,17,18,19]. Their rates can be increased through various reactions such as abiotic hydrolysis, photo-oxidation, and physical disintegration [20]. Various fungal genera have been reported previously in the biodegradation of plastics, such as *Gliocladium*, *Cunninghamella*, *Penicillium*, *Aspergillus*, *Fusarium*, *Mucor*, and *Mortierella* [21,22,23]. In addition, many filamentous fungi including *A. niger*, *Aspergillus terreus*, *Aureobasidium pullulans*, *Paecilomyces varioti*, *Penicillium funiculosum*, *Penicillium ochrochloron*, *Scopulariopsis brevicaulis*, and *Trichoderma viride* were reported to have LDPE-degrading abilities [24]. The ability of fungi in the biodegradation of plastics was enhanced by producing special intracellular and extracellular enzymes for degrading polymers into small oligomers, dimers, and monomers [4,14]. These products are used as carbon sources for fungi growth and are converted into water, carbon dioxide, or methane [25]. Thus, the discovery of the biodiversity and occurrence of fungi with the potential to effectively biodegrade plastics has been a challenge for researchers.

Recently, the biodiversity of more than 500 species from both macro- and micro-fungi in Thailand was reported [26]. These species were discovered as microfungal plant pathogens, saprobes, endophytes, and epitypes [27]. Many thousands of strains of fungi from Thailand have been isolated and studied over the past few years [28]. Most of them have never been explored for their biodegradation potential. Accordingly, the obtained species of Thai mycobiota may be good candidates for the discovery of fungi for degrading plastics. Therefore, this study presents the LDPE biodegradation potential of fungal species from Thailand. The study also focuses on the biodegradation efficiency evaluated by FTIR, SEM, and GC-MS. The production of CO_2_ and enzymes after incubation was also determined.

## 2. Materials and Methods

### 2.1. Fungal Strains

Thirty fungal species were selected for this study, including *Ophiosimulans tanaceti*, *Populacrescentia foricesenensis*, *Embarria clematidis*, *Sclerostagonospora lathyri*, *Pseudomassariosphaeria bromicola*, *Nodulosphaeria multiseptata*, *Vagicola chlamydospore*, *Halobyssothecium obiones*, *Paraepicoccum amazonense*, *Poaceascoma halophila*, *Pestalotiopsis microspore*, *Muyocopron heveae*, *Muyocopron dipterocarpi*, *C. fructicola*, *Collectotrichum pandanicola*, *Alternaria burnsii*, *Setoseptoria arundelensis*, *Cytospora centravillosa*, *D. italiana*, *Coniella vitis*, *Septomelanconiella thailandica*, *Murilentithecium lonicerae*, *Phragmocamarosporium hederae*, *Keissleriella caraganae*, *Neomollisia gelatinosa*, *T. jaczewskii*, *Tamsiniella labiosa*, *Diaporthe rumicicola*, *Epicoccum pseudokeratinophilum*, and *S. citrulli*. These strains were obtained from the culture collection of the Institute of Excellence in Fungal Research, Mae Fah Luang University, Thailand. The reference fungal strain used in this study was *A. niger* ATCC 10254, which was acquired from the culture collection of the Thailand Institute of Scientific and Technological Research, Bangkok, Thailand.

### 2.2. Preparation of LDPE Film

White and amorphous LDPE microplastic granules, 5 mm nominal size, (Sigma-Aldrich, Singapore) were used in this study. The LDPE film was prepared according to a modified method [29]. The 0.12 mm polymeric film was prepared by melting 0.2 g of LDPE polymer between aluminum foil in a hydraulic press (Carver, Wabash, IN, USA) at a temperature of 180 °C and under 55.3 kgf/cm^2^ of pressure for 5 min. The obtained films were cooled at room temperature.

### 2.3. Screening of Potential Fungi for LDPE Film Degradation

All fungal strains described above were screened for their ability to degrade LDPE films. The biodegradation test was performed in mineral salt medium broth containing LDPE film as the only sole carbon source [24]. The mineral salt medium broth containing 3 g NH_4_NO_3_, 5 g K_2_HPO_4_, 1 g NaCl, 0.2 g MgSO_4_·7H_2_O, and 1000 mL of deionized water was prepared following the study of Brunner et al. [30]. All chemicals are purchased from Sigma-Aldrich (Singapore). The medium contained the nutrients including nitrogen, phosphorus, sulfur, potassium, magnesium, sodium, and chlorine. A LDPE film (1.0 cm × 4.0 cm) was placed into 250 mL of mineral salt medium broth in a 1000 mL Erlenmeyer flask prior to the inoculation of five agar plugs (5 mm diameter, 7 days old) of each strain. All solution flasks were stored at pH 7.0 ± 0.2. Fungal cultures were incubated at room temperature (27.0 ± 2.0 °C) for 30 days under static conditions. The fungus *A. niger* was used as the control to confirm the degradation of LDPE film. After 30 days, fungal mycelium was filtered and vacuum dried for 24 h. Mycelium dry weight obtained from each fungus was measured by digital weighing balance.

### 2.4. Culturing Condition

LDPE films were placed in mineral salt medium broth with agar plugs of potential fungi, as described above. The cultures were incubated at room temperature (27.0 ± 2.0 °C) under static conditions for a duration of 30, 60, and 90 days. The fungus *A. niger* (control) was tested as a basis for comparison.

### 2.5. Screening of Enzymes Production

The enzyme activity of potential fungi cultured with LDPE film after 30, 60, and 90 days of incubation was investigated following the study of Ameen et al. [31]. After the incubation period, the culture supernatant was separated from fungal culture and the obtained filtrates were tested for their enzyme activity, including laccase, manganese peroxidase, and lignin peroxidase. All chemicals were obtained from Sigma Chemical Co., St. Louis, MO, USA. Enzymic activity of laccase was determined at 420 nm via the oxidation of 2,2-azino-bis-3-ethyl-benzthiazoline-6-sulfonic acid (ABTS). Culture supernatant (50 µL) was mixed with 0.1 mM ABTS and 100 mM sodium tartrate (pH 4.5). Enzymic activity of manganese peroxidase was determined at 238 nm by mixing 50 µL of culture supernatant with 0.01% phenol in 0.1 mM H_2_O_2_ and 1 mM MnSO_4_ in 100 mM sodium tartrate (pH 4.5). Enzymic activity of lignin peroxidase was determined at 310 nm by the oxidation of 2 mM veratryl alcohol in 100 mM sodium tartrate (pH 4.5) with 0.4 mM H_2_O_2_ and 50 µL of culture supernatant. One unit (U) of enzymic activity was defined as the amount of enzyme required to oxidize 1 µmol of product per min at 30 °C and pH 4.5. The enzymic activity obtained from all enzymes were expressed as U/mL.

### 2.6. Evaluation of CO_2_ Production

The amount of CO_2_ released by potential fungi culturing with LDPE film was determined using a modified sturm test. Sterile air was firstly flowed via 1 M KOH solution to eliminate atmospheric CO_2_ prior culturing. Dissolved CO_2_ in the culture broth was determined by the titration method [31] after 30, 60, and 90 days of incubation at room temperature (27.0 ± 2.0 °C) with static conditions. The broth of each fungus was filtered, and 25 mL of filtrate was mixed with 0.05 mL of 0.1 N Na_2_S_2_O_3_ and 2 drops of methyl orange in an Erlenmeyer flask. The solution was titrated with 0.02 M NaOH solution until a yellow color was observed. After that, two drops of phenolphthalein were added into the flask and titration was performed until a pink color was observed. Volumes of the used titrant were recorded. The CO_2_ amount in the broth was calculated using the following equation: [A × B × 50 × 1000]/V, where A = volume of NaOH in mL, B = normality of NaOH, and V = volume of sample in mL. All chemicals are purchased from Sigma-Aldrich (Singapore).

### 2.7. Characterization of LDPE Films by the Potential Fungal Strains

After the incubation periods, the LDPE films were removed from the fungal culture and rinsed with 70% ethanol and sterile distilled water [32]. The obtained LDPE films from each fungal culture were air dried at ambient temperature for 24 h prior to analysis. The weight of each recovered film was recorded using analytical balances. The percentage of weight loss was calculated using the formula [24] % weight loss = [(initial weight − final weight)/initial weight)] × 100. The tensile strength of the LDPE films following degradation tests were also determined using a Universal Testing Machine (UTM; 8800 servo-hydraulic testing machine, Instron, Kawasaki, Japan) in displacement-controlled mode according to the modified method of Amjadi and Fatem [33]. Two dots with a distance of 3 mm apart in the gage section of the LDPE films were marked. Tension strength was measured between these two marked points. Furthermore, the topography of LDPE films obtained after 30, 60, and 90 days of fungal treatment were analyzed using scanning electron microscopy (SEM; Hitachi SU-70). The dehydrated samples were sputter-coated with gold prior to SEM analysis [25]. The sputtering was performed after placing the pure and dry argon gas in the coating chamber under vacuum. The 10 kV and the 40 mA current were employed in the system. A thickness of 2 nm of gold was used during the 45 s sputtering time. The sample was then examined under the SEM. The chemical structures of LDPE films obtained after 30, 60, and 90 days of incubation were characterized using FTIR (Lumos, Bruker, Billerica, MA, USA) with 64 scans at a resolution of 4 cm^−1^ for each sample [24]. All spectra presented in the results are obtained in the wavenumber range of 600–4000 cm^−1^. Moreover, the carbonyl index value was calculated as CI. CI is defined as the ratio between the integrated band absorbance of the carbonyl functional group peak from 1850 to 1650 cm^−1^ to those obtained from the methylene (CH_2_) scissoring peak from 1500 to 1420 cm^−1^. The CI values were calculated using the following equation: CI = area under band 1850–1650 cm^−1^/area under band 1500–1420 cm^−1^ [34].

### 2.8. GC-MS Analysis

After 30, 60, and 90 days of incubation, 10 mL of the broth of each fungus was collected and filtered. VOCs in the obtained supernatant were extracted with 10 mL of dichloromethane (Sigma-Aldrich, Singapore) using a separatory funnel following a modified method from Park and Kim [35]. VOCs emitted from the LDPE film were analyzed using the GC-MS system (Agilent Technologies, Santa Clara, CA, USA). The capillary column was an HP-5ms (30 m × 0.25 mm i.d., 0.25 µm film thickness) (Agilent Technologies, Santa Clara, CA, USA). The oven temperature was initially held at 60 °C and then increased at a rate of 5 °C/min to a final temperature of 220 °C. The injector temperature was set to 250 °C. Helium was used as a carrier gas with a flow rate of 1 mL/min. Electron impact mass spectra were collected at 70 eV over the range of 30–300 *m*/*z*. The ion source and analyzer temperatures were set to 250 and 230 °C, respectively. Identification of VOCs was performed by comparing their mass spectra with those found in the NIST14 Mass Spectral Library.

### 2.9. Statistical Analysis

All experiments were performed in triplicate five times. Data from each experiment were presented as the mean ± standard deviation. Data were subjected to Analysis of variance (ANOVA) followed by post hoc multiple pairwise comparisons using Duncan’s multiple range tests with a reference treatment (α = 0.05). The statistical analyses were conducted using the SPSS 20.0 software (IBM Corp). The data for each bar in all figures are mean ± standard deviation. Different letters above the bars indicate significant differences (*p* < 0.05) among the samples (ANOVA, followed by Duncan’s multiple range test).

## 3. Results

### 3.1. Screening of Potential Fungi for LDPE Film Degradation

**Figure 1:**
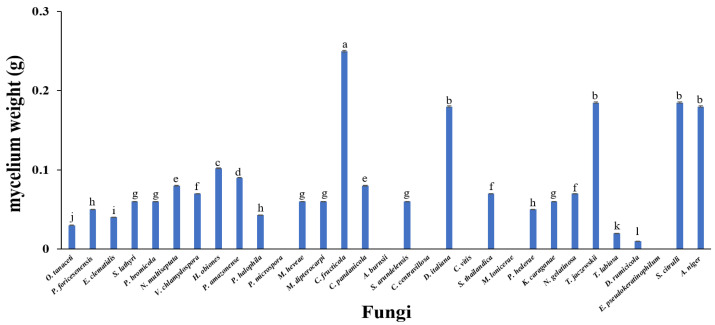
Mycelium dry weight of fungi cultured with LDPE film after 30 days of incubation. Different letters above the bars indicate significant differences (*p* < 0.05).

All fungal strains as described above were tested for their ability to degrade LDPE films as a carbon source on a liquid medium. The growth ability of fungi culturing with LDPE film after 30 days of incubation was evaluated as mycelium dry weight of the fungi (Figure 1). The mycelium dry weight of fungi varied from 0 to 0.25 g. Five efficient fungi, including *D. italiana*, *T. jaczewskii*, *C. fructicola*, *S. citrulli* and *A. niger*, were able to grow significantly in media, showing a mycelium dry weight of 0.25, 0.18, 0.19, 0.19, and 0.18 g, respectively, compared to those obtained from other fungi. Therefore, these fungi were further analyzed for their degradation ability.

### 3.2. Enzyme Activity of Potential Fungi

**Figure 2:**
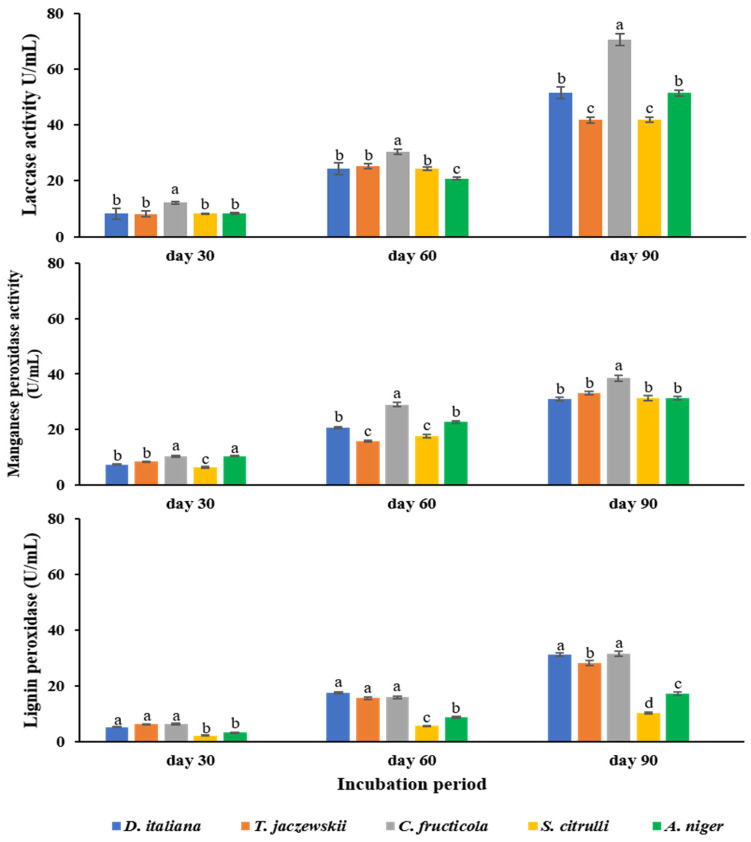
Enzymic activity in *D. italiana*, *T. jaczewskii*, *C. fructicola*, *S. citrulli*, and *A. niger* culture supernatant after 30, 60, and 90 days of incubation. Different letters above the bars indicate significant differences (*p* < 0.05).

The enzymatic activity of laccase, manganese peroxidase, and lignin peroxidase secreted by potential fungi are depicted in Figure 2. All fungi were able to secrete all enzymes. The enzymic activity was enhanced when the incubation period was increased. However, enzyme laccase was highly secreted by all fungi compared to other enzymes in the same conditions. The *C. fructicola* fungus secreted the highest level of laccase after 90 days of incubation, with 70.67 U/mL, while other fungi secreted laccase ranging from 41.78 to 51.56 U/mL. Enzyme manganese peroxidase was detected with a similar content in all fungi, ranging from 6.33 to 10.37, 15.75 to 22.77, and 31.11 to 38.45 U/mL after 30, 60, and 90 days of incubation, respectively. In addition, lignin peroxidase was also detected in all fungi, ranging from 2.33 to 6.45, 5.68 to 17.66, and 10.26 to 31.45 U/mL after 30, 60, and 90 days of incubation, respectively.

### 3.3. CO_2_ Production from Potential Fungi

**Figure 3:**
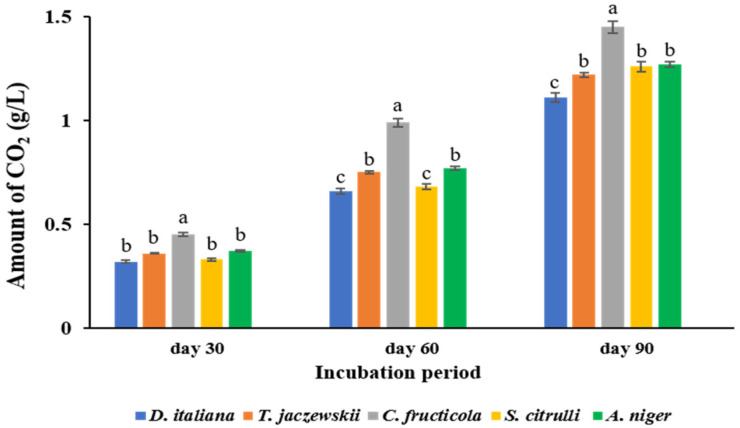
Amount of CO_2_ dissolved in *D. italiana*, *T. jaczewskii*, *C. fructicola*, *S. citrulli*, and *A. niger* broth after 30, 60, and 90 days of incubation. Different letters above the bars indicate significant differences (*p* < 0.05).

The amount of CO_2_ dissolved in the culture broth of *D. italiana*, *T. jaczewskii*, *C. fructicola*, *S. citrulli*, and *A. niger* after 30, 60, and 90 days of incubation is shown in Figure 3. The fungus *C. fructicola* demonstrated the highest amount of CO_2_ compared to other fungi, ranging from 0.45 to 0.99, and 1.45 g/mL after 30, 60, 90 days of incubation, respectively. Similar CO_2_ amounts were detected in broths of *D. italiana*, *T. jaczewskii*, *S. citrulli*, and *A. niger* ranging from 0.36 to 1.22, 0.45 to 1.45, 0.33 to 1.26, and 0.37 to 1.27 g/L, respectively, after 90 days of incubation.

### 3.4. Determination of Weight Loss of LDPE Films

**Figure 4:**
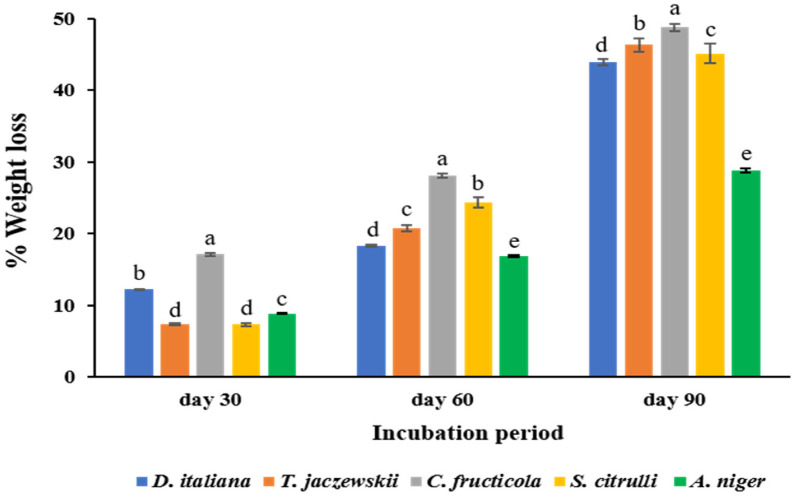
Weight loss percentage of LDPE films cultured with *D. italiana*, *T. jaczewskii*, *C. fructicola*, *S. citrulli*, and *A. niger* after 30, 60, and 90 days of incubation. Different letters above the bars indicate significant differences (*p* < 0.05).

Biodegradation of LDPE films by culturing with culture medium of *D. italiana*, *T. jaczewskii*, *C. fructicola*, *S. citrulli*, and *A. niger* was evaluated after 30, 60, and 90 days of incubation (Figure 4). It was found that the weight loss of LDPE films during the incubation with the five efficient fungal strains was resulted from the utilization of the polyethylene as the sole carbon source. The results indicate that these fungal strains were capable of degrading LDPE films, and that the fungus *C. fructicola* showed the highest LDPE film degradation ability, with 17.07%, 28.05%, and 48.78% after 30, 60, and 90 days of incubation, respectively. The weight loss percentages of LDPE films cultured with broths of the *D. italiana* fungus were 12.20%, 18.29%, and 43.90% after 30, 60, and 90 days of incubation, respectively. Similarly, the percentage of weight loss of LDPE films was also increased after culturing with culture broths of *T. jaczewskii*, with 7.32%, 20.73%, and 46.34% after 30, 60, and 90 days of incubation, respectively. LDPE films from *S. citrulli* culture revealed weight loss percentages of 7.32%, 24.39%, and 45.12% after 30, 60, and 90 days of incubation, respectively. The LDPE films from the *A. niger* culture showed weight loss percentages of 8.85%, 16.83%, and 28.78% after 30, 60, and 90 days of incubation, respectively.

### 3.5. Determination of Tensile Strength of LDPE Films

**Figure 5:**
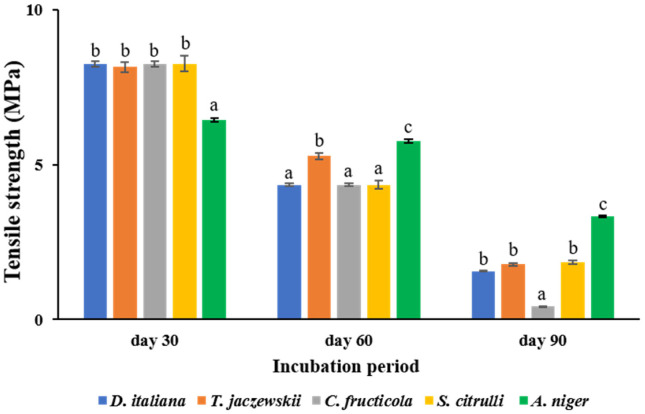
Tensile strength of LDPE films cultured with *D. italiana*, *T. jaczewskii*, *C. fructicola*, *S. citrulli*, and *A. niger* after 30, 60, and 90 days of incubation. Different letters above the bars indicate significant differences (*p* < 0.05).

The tensile strength values of LDPE films cultured with *D. italiana*, *T. jaczewskii*, *C. fructicola*, *S. citrulli*, and *A. niger* after 30, 60, and 90 days of incubation are shown in Figure 5. *C. fructicola* was able to reduce the tensile strength of LDPE films at the highest rate after incubation of 30, 60, and 90 days of incubation to 8.26, 4.35, and 0.43 MPa, respectively. The strength of the LDPE films was reduced significantly to 8.26 MPa, 4.35, and 1.56 MPa after culturing with *D. italiana* after 30, 60, and 90 days of incubation. The fungi *T. jaczewskii* also showed a significant decrease in tensile strength after 30, 60, 90 days of incubation to 8.16, 5.28, 1.78 MPa, respectively. The fungus *S. citrulli* revealed tensile strengths of LDPE films of 8.26, 4.35, and 1.86 MPa, respectively, whereas tensile strengths of LDPE films of 6.45, 5.77, and 3.34MPa were determined in LDPE films culturing with *A. niger* after 30, 60, and 90 days of incubation. The percentage of tensile strength of LDPE films culturing with *D. italiana*, *T. jaczewski*, *C. fructicola*, *S. citrulli*, and *A. niger* was reduced to 6.40%, 5.61%, 23.21%, 5.37%, and 2.99%, respectively, after 90 days of incubation. This indicates that LDPE films cultured with these fungal cultures were fragile.

### 3.6. FTIR and the CI Analysis of the LDPE Films

**Figure 6:**
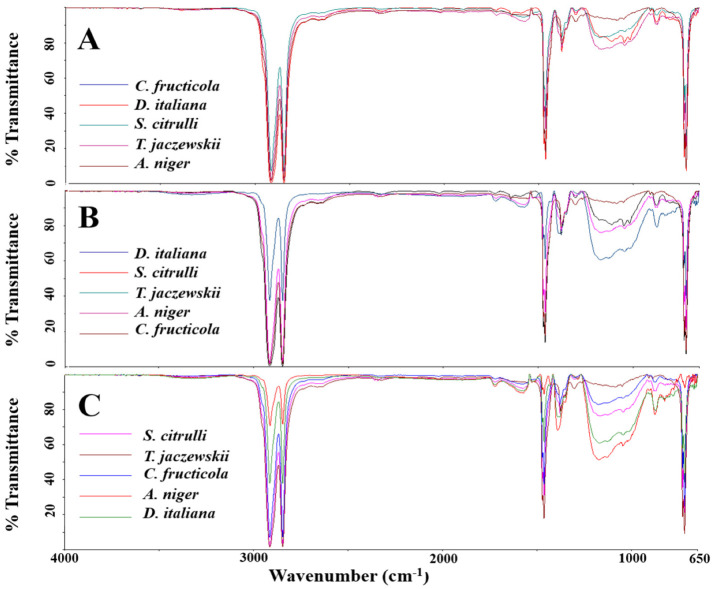
Spectra from FTIR analysis of LDPE films cultured with *D. italiana*, *T. jaczewskii*, *C. fructicola*, *S. citrulli*, and *A. niger* after 30 (**A**), 60 (**B**), and 90 (**C**) days of incubation.

The LDPE films cultured with culture broths of *D. italiana*, *T. jaczewskii*, *C. fructicola*, *S. citrulli*, and *A. niger* after 30, 60, and 90 days were analyzed by FTIR, and the spectra are shown in Figure 6. The absorption strong band for aliphatic CH was observed at 2954 and 2856 cm^−1^ in the spectra of all samples. Characteristic bands of low intensity at 1740, 1624, and 1546 cm^−1^ depicted in LDPE films were obtained from the spectra of these fungi. A quite low intensity of the broad band at 3370 cm^−1^ was also detected in LDPE films obtained after culturing with these fungal culture broths. Medium bands at 1470 and 1420 cm^−1^ were assigned to CH_3_ deformation, and 1228 and 1055 cm^−1^ to C-O-C stretching were also detected in the spectra of LDPE films obtained from all fungi. Similarly, all LDPE films showed absorbance in the range of 700–900 cm^−1^ corresponding to -C=C- stretching and the presence of alkene groups.

**Figure 7:**
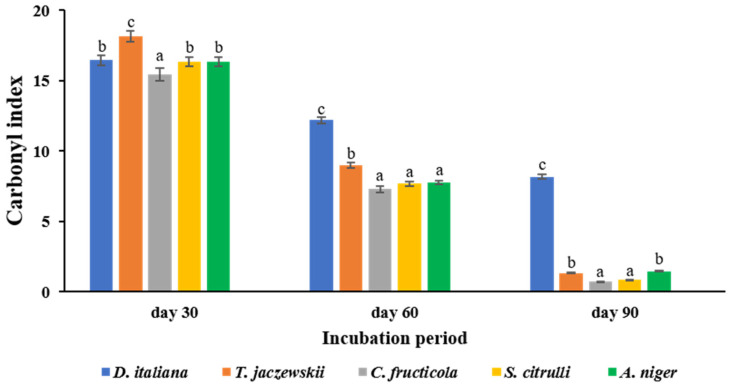
The carbonyl indexes of the of LDPE films cultured with *D. italiana*, *T. jaczewskii*, *C. fructicola*, *S. citrulli*, and *A. niger* after 30, 60, and 90 days of incubation. Different letters above the bars indicate significant differences (*p* < 0.05).

The CI of the LDPE films by culturing with culture broths of *D. italiana*, *T. jaczewskii*, *C. fructicola*, *S. citrulli*, and *A. niger* after 30, 60, and 90 days of incubation were also calculated and depicted in Figure 7. The decrease in the CI was determined to confirm the oxidation of LDPE films by the tested fungi. The CIs for the LDPE films when culturing with *D. italiana*, *T. jaczewskii*, *C. fructicola*, *S. citrulli*, and *A. niger*, after 30, 60, and 90 days of incubation were 16.45–8.18, 18.16–1.34, 15.45–0.74, 16.33–0.86, and 16.37–1.47, respectively.

### 3.7. Characterization of the LDPE Films by SEM Analysis

**Figure 8:**
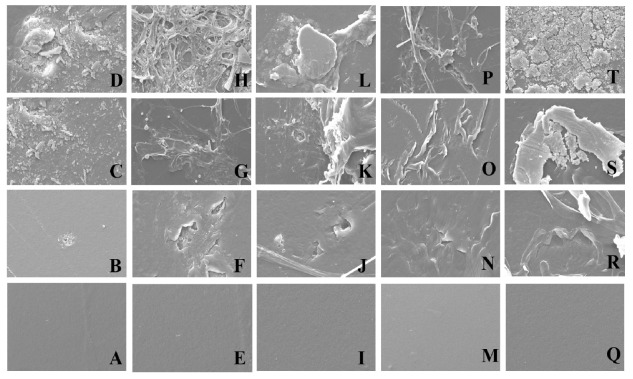
Scan electron micrographs of LDPE films (1000×) cultured with *D. italiana* (**A**–**D**), *C. fructicola* (**E**–**H**), *T. jaczewskii* (**I**–**L**), *S. citrulli* (**M**–**P**), and *A. niger* (**Q**–**T**) after 0, 30, 60, and 90 days of incubation, respectively.

To confirm the biodegradation of LDPE films by the tested fungi, the LDPE films were analyzed by SEM. The surface morphology of the LDPE films cultured with culture broths of *D. italiana*, *T. jaczewskii*, *C. fructicola*, *S. citrulli*, and *A. niger* after 30, 60, and 90 days of incubation are demonstrated in Figure 8. In the case of *D. italiana*, *T. jaczewskii*, *C. fructicola*, *S. citrulli*, and *A. niger*, various structural changes such as grooves, cracks, damaged layer, fragileness, pits and roughening of the surface were observed after 30, 60, and 90 days of incubation. No apparent structural changes were found on day 0. In the case of LDPE films treated by the fungus *C. fructicola*, attachment of the spores was observed after 60 and 90 days of incubation.

### 3.8. GC-MS Analysis

**Figure 9:**
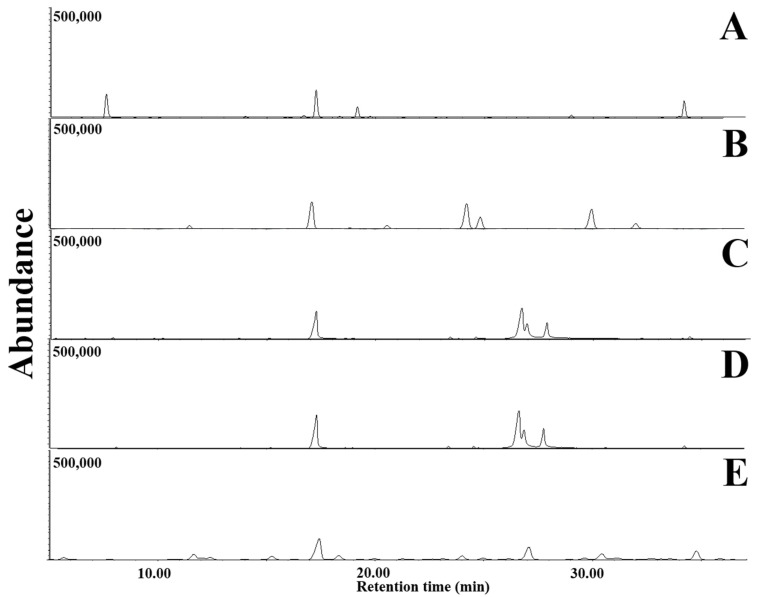
GC-MS chromatograms of dichloromethane extracts of LDPE films cultured with *T. jaczewskii* (**A**), *S. citrulli* (**B**), *A. niger* (**C**), *D. italiana* (**D**), and *C. fructicola* (**E**) after 30 days of incubation, respectively.

**Figure 10:**
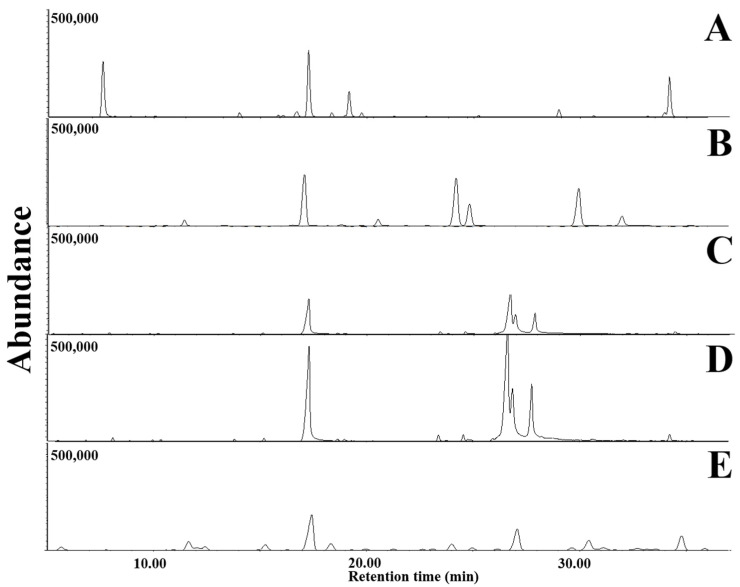
GC-MS chromatograms of dichloromethane extracts of LDPE films cultured with *T. jaczewskii* (**A**), *S. citrulli* (**B**), *A. niger* (**C**), *D. italiana* (**D**), and *C. fructicola* (**E**) after 60 days of incubation, respectively.

**Figure 11:**
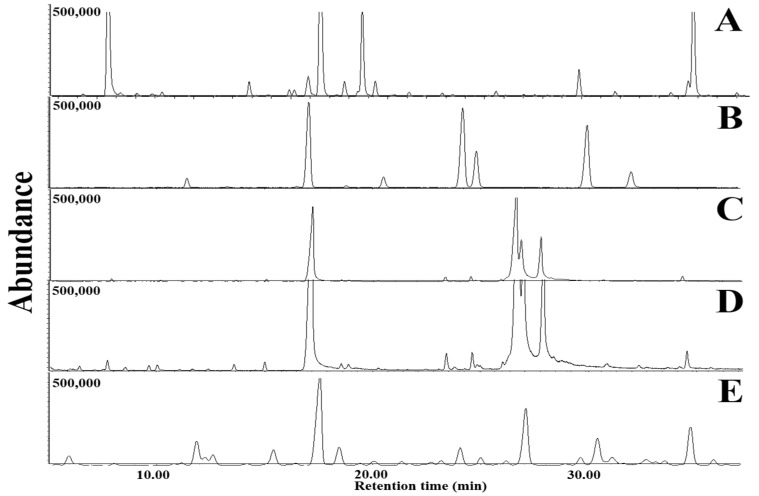
GC-MS chromatograms of dichloromethane extracts of LDPE films cultured with *T. jaczewskii* (**A**), *S. citrulli* (**B**), *A. niger* (**C**), *D. italiana* (**D**), and *C. fructicola* (**E**) after 90 days of incubation, respectively.

VOCs emitted from LDPE films culturing with *D. italiana*, *T. jaczewskii*, *C. fructicola*, *S. citrulli*, and *A. niger* after 30, 60, and 90 days of incubation was determined using GC-MS analysis. GC-MS chromatograms of VOCs emitted from LDPE films after 30, 60, and 90 days of incubation are shown in Figure 9, Figure 10 and Figure 11, respectively. The number of VOCs and peak height increased with the increasing incubation period. A total of 27 VOCs was detected among these samples obtained from 90 days of incubation. The quantitative result is shown in Table 1. The highest number of VOCs (22 compounds, representing 98.85% of the total amount) was detected from *C. fructicola* when compared with those emitted from *T. jaczewskii* (21 compounds, representing 99.85% of the total amount), *A. niger* (15 compounds, representing 98.77% of the total amount), *D. italiana* (15 compounds, representing 96.87% of the total amount), and *S. citrulli* (8 compounds, representing 98.77% of the total amount).

Undecanal, phenyl-4-methyl-pentan-3-one, and phenyl pentan-3-one were found to be the major compounds in LDPE film culturing with *T. jaczewskii*. Meanwhile, phenyl-4-methyl-pentan-3-one, 1,3-dimethoxy-benzene, and 1,3-dimethoxy-5-(1-methylethyl)-benzene were detected in high amounts in LDPE film culturing with *S. citrulli*. Similar major compounds including 1,1-dimethoxy-decane, 1,3-dimethoxy-benzene, and 2E,6Z-diethylacetal-nonadienal were detected in LDPE film culturing with *A. niger* and *D. italiana*, while 1,3-dimethoxy-benzene, 1,1-dimethoxy-decane, and 2-dodecanone were considered as major compounds in LDPE film culturing with *C. fructicola*.

## 4. Discussion

This study consists of both saprotrophic and plant pathogenic fungi such as *O. tanacetiand*, *E. clematidis*, *M. heveae*, *S. citrulli*, *T. jaczewskii*, and *H. obiones* [26,27,36]. Some fungi are known as plant pathogens, such as *C. fructicola* [37], and *D. italiana* [38]. The degradation ability of fungi cultured with the culture broths was depicted via weight loss percentage, tensile strength, formation of cracks/scars/holes on the surface of the LDPE films, and changes in the CI [5,39]. This is the first study that demonstrates the degradation of LDPE films by fungi obtained from Thailand. From the preliminary screening, four fungal species showed degradation potential: *D. italiana*, *T. jaczewskii*, *C. fructicola*, and *S. citrulli*, compared to *A. niger*, which has been reported to have degradation ability [40,41]. The different degradation potential of all tested fungi may be mainly influenced by various other factors, such as incubation period, temperature, humidity, carbon source, and pH [39,42].

The efficiency of degradation by active fungi was further confirmed using liquid medium with which the carbon sources for the growth of fungi were limited. The growth rate of each fungus on the surface of LDPE films in the liquid medium varied at room temperature. This result may be because the degradation temperature plays an important role in biodegradation [40,41,42]. Although several reports presented the potential of *A. niger* for LDPE degradation [39,40,41,42,43], the active fungi in this study showed significantly higher potential during the same incubation period.

These potential fungi were able to secrete different enzymes in terms of qualitative and quantitative analysis. This result may be dependent on genotype in nature [31]. Recently, biodegradation of LDPE polymer was enhanced by extracellular enzymes produced by the potential microorganism [31,35]. Therefore, enzyme levels in broths of these potential fungi indicated the degenerative activity. The level of the enzyme laccase was higher in all cultures growing with LDPE film, probably due to the greater demand for polymer breakdown reactions. The fungus *C. fructicola* showed a remarkably higher level of the enzyme laccase as compared to other fungi. This may be due to great contribution with biomass accumulation. Several polyethylene degrading enzymes were reported such as phylloplane fungal enzymes from *Paraphoma*-like phylloplane fungus [44], laccase, and manganese peroxidase from *Penicillium simplicissimum* [45]. Some fungi have been evaluated to secrete the enzyme depolymerase, which is responsible for hydrolyzing and breaking polymers into oligomers and monomers such as *Penicillium pinophyllum* [44], *Paecilomyces lilacinus* [46], and *Fusarium moniliforme* [47]. The products of the reaction were then further transformed into water and carbon dioxide. However, the degradation mechanism of fungi is not exactly known. Gu et al. [48] reported that at least two categories of enzymes, including extracellular and intracellular depolymerases, were mainly related in the depolymerization process of polymer degradation. During the degradation process, extracellular enzymes from fungi break down complex polymers into oligomers, dimers, and monomers. These products are sufficiently small and can be further utilized as carbon sources [44,45]. Their mechanism involved in biodegradation pathways will be further studied for searching for new or alternative technologies for degrading these environmentally hazardous LDPE films.

In general, CO_2_ released by fungi culturing with LDPE film was also evaluated as a reliable indicator of biodegradation ability [49]. During the incubation period, these potential fungi enzymatically degraded polymer chains of LDPE films and released CO_2_. These potential fungi showed a great amount of CO_2_ after the incubation period, demonstrating degradation ability. Pramila and Ramesh [50] reported that higher amount of CO_2_ was released by *Aspergillus* species culturing with LDPE films. Shah et al. [51] revealed a great amount of CO_2_ in a *Fusarium* culture from sewage waste culturing with polyethylene bag pieces. Esmaeili et al. [42] reported that the amount of CO_2_ was significantly higher in treatments supplemented with the potential fungi.

High weight loss of LDPE films obtained from culturing with the culture broths may be due to the breakdown of its carbon backbone via enzymatic degradation processes by the fungi, thereby producing monomers and oligomers as a carbon source [52]. The tensile strength, one of the main parameters of LDPE films, was also determined. It was found that a strong correlation between weight loss percentage and tensile strength was depicted. The highest weight loss and lowest tensile strength were shown in the LDPE films cultured with culture broths of *C. fructicola*, followed by those of *T. jaczewskii*, *S. citrulli*, and *D. italiana*, respectively. Our report is in good agreement with the study by Vijaya and Reddy [53]. They reported the correlation coefficient between weight loss and tensile strength. A strong correlation coefficient was achieved when weight loss was affected by microbial attacks, and that tensile strength also affected at the same time.

In the present study, the FTIR data confirm changes in bond scission, chemical transformation, formation, and disappearance of new functional groups in the LDPE films. These changes indicate that the LDPE films may be degraded by the fungal enzymes, resulting in the breakdown and formation of some chemical bonds [4,5,54]. For example, 3-poly hydroxybutyrate depolymerase (PHB), it was secreted by *Penicillium pinophyllum* [24] and responsible for hydrolyzing of ester bonds in polyethylene and breaking it into oligomers and mono (3 hydroxybutyrate). The changes in the detected spectrum were correlated with band intensity and the possible displacement of these bands or their appearance [55]. The degradation rate was increased with incubation time. The peaks were broader as various monomeric forms and oxidation products of the LDPE films were generated [56]. No significant changes were observed among LDPE films obtained from culturing with these fungal cultures. However, some changes were present, such as the bands at 1740 cm^−1^ (C=O groups stretching in the amorphous phase) and 1720 cm^−1^ (C=O groups stretching in the crystalline phase), and the new bands appearing at 1624 and 1546 cm^−1^ correspond to C=O acid and C=C vibration bands, respectively. The appearance of the band at 3370 cm^−1^ (broad band) corresponds to the O-H vibration of acids. These changes could be attributed to the presence of organic carboxylic acid produced from hydrolysis reactions by fungal enzymes [57]. The significant bands at 1470, 1420, 1228, and 1055 cm^−1^ obtained from CH_3_ deformation and C-O-C stretching were sensitive to the polymer crystallization which decreases the band intensity and film crystallinity in the C-O-C ester group [58]. Bands at 700–900 cm^−1^ corresponding to -C=C- stretching and the presence of alkene groups found in the LDPE may also be generated from hydrolysis reactions by fungal enzymes [42]. FTIR analysis shows that the LDPE films were degraded significantly by the fungus *C. fructicola* possibly via the enzymatic hydrolysis reaction promoting the microbial action. The CI value, which determines the degradation rate, decreased when the incubation time was increased and was observed to be the lowest for the fungus. The decreased CI confirmed the concept that oxidized polymers are utilized by the fungi [59]. In addition, Pathak [60] reported that the degradation rate of polymers depends on the time required for the growth phase of fungal culture and the addition of functional groups from VOCs emitted from LDPE films.

The degradation of the LDPE films was further confirmed using SEM analysis. The SEM analysis revealed the degradation level on the surface of the LDPE films in the form of cracks, scions, and holes. The SEM images confirmed that these five fungal strains were able to break down the complex structure of polyethylenes of LDPE into their monomeric forms. The cracks also confirmed the fragility of LDPE films cultured with these fungal cultures. The degradation was enhanced by the formation of biofilms by fungi as reported by Das and Kumar [61]. The fungal strains were colonized on the LDPE film by utilizing their film as the sole carbon source, and the fungal hyphae have successfully been coalesced with LDPE film by growing over the film surface. Our SEM analysis results were similar to previous reports exhibiting structural changes and erosions on the surface of LDPE films in terms of porosity, cavities, holes, scions, and cracks by consortia of some fungi such as *Aspergillus* and *Penicillium* [40,61]. The surface of LDPE films obtained from culturing with these fungal cultures was changed from smooth to holes with cracks, and a decrease in molecular weight and an increase in carbonyl double bond groups caused by the fungi were also observed.

VOCs emitted from LDPE films culturing with potential fungi were identified using GC-MS. Most VOCs in all extracts were major hydrocarbons originated from polymer chains in LDPE films with functional groups of C-O-C and -C=C- detected from the FTIR analysis. These compounds might be produced from oligomers generated during the LDPE polymerization [35]. These VOCs may be produced from multiple pathways, including the primary, secondary and tertiary radicals [62]. In addition, some VOCs with oxygen-containing compounds might be generated from oxidation degradation of LDPE during incubation [63]. The degradation of LDPE chain by each fungus was dependent on degradation processes with different multiple active sites of the catalyst or mechanical degradation products [63]. The results show that the degradation of LDPE films by different fungi produce particular VOC emissions, the levels of these emissions have been demonstrated to change with increased degradation. Analysis of these VOCs showed evidence of degradation and enabled degradation progress to be monitored. VOC analysis could lead to improved condition assessment and monitoring of degradation of a LDPE films. However, mechanisms responsible for VOC emission from polymers are unknown.

The fungi collected from Thailand were successful in degrading LDPE films under laboratory conditions, especially *C. fructicola*. Their effectiveness in the degradation of LDPE films by culturing was investigated over an incubation period of 90 days. It is noted that releasing of CO_2_ and enzyme production by potential fungi lead to higher LDPE degradation values. In addition, FTIR analysis showed the formation of carbonyl groups, CH stress, and CH_2_ groups. CI index from FTIR analysis was decreased after culturing with fungi. These decreasing values are due to the consumption of carbonyl and carboxylic acid derivatives by fungi, indicating the depolymerization of the polymeric chain. Analysis through SEM and GC-MS also confirmed the degradation of LDPE films, showing the presence of erosion and fragility of the LDPE film surface. The degradation mechanism may be attributed to enzyme production by fungal actions. Therefore, the obtained information can be used as evidence for the degradation potential of the fungi on LDPE films which can be further applied at an industrial scale for degrading various plastic materials.

However, there is limited information about the optimum conditions, such as incubation period, temperature, humidity, inorganic medium and pH, for culturing potential fungi. In addition, the determination of toxicity level after culturing is also necessary before using them as a biodegradable fungus.

## Figures and Tables

**Table 1:** VOCs emitted from LDPE films cultured with *T. jaczewskii*, *S. citrulli*, *A. niger*, *D. italiana*, and *C. fructicola* after 90 days of incubation.

No.	Compound	*T. jaczewskii*	*S. citrulli*	*D. italiana*	*C. fructicola*	*A. niger*
1	hexadienal	1.81		0.78		0.82
2	methyl-1,4-cyclohexadiene	10.21		0.25	6.21	0.23
3	octene	1.67		1.23		0.58
4	3,3,5-trimethyl-cyclohexene	2.45		0.21	0.11	0.23
5	3,5,5-trimethyl-cyclohexene	2.57		0.31	0.11	0.31
6	2E-methyl-3-octen-5-yne			1.12	0.34	1.09
7	1,2,3-trimethyl benzene		3.23		4.34	
8	decene			0.48	0.12	0.59
9	decane			0.86	1.19	0.98
10	1,2,4-trimethyl benzene	3.87				
11	2-methoxyethyl-benzene	0.21		0.79	0.98	0.84
12	pentyl-benzene	1.89			3.45	
13	1,4- dimethoxybenzene	0.86	0.11		0.32	
14	1,3-dimethoxy-benzene	2.67	18.48	21.32	32.01	23.54
15	Undecanal	22.21			5.37	
16	undec-9Z-en-1-al	1.21			0.23	
17	phenyl pentan-3-one	12.23	3.43		0.63	
18	1,3-dimethoxy-5-(1-methylethyl)-benzene	4.11	17.22	1.34	4.34	1.48
19	trimethyl benzaldehyde	1.91				
20	2E-undecenal	0.24	14.58	1.42	3.19	1.31
21	1,1-dimethoxy-decane	1.02		50.13	17.56	51.11
22	2E,6Z-diethylacetal-nonadienal			15.34		14.38
23	phenyl-4-methyl-pentan-3-one	15.35	24.76		0.18	
24	hexyl hexanoate				7.01	
25	3Z-hexenyl-3Z-hexenoate	1.13	15.34		0.88	
26	isobutyl phenylacetate	2.21		1.29		1.28
27	2-dodecanone	10.02			10.28	
	Total of % area	99.85	97.15	96.87	98.85	98.77
	No. of VOCs	21	8	15	22	15

## Data Availability

Not applicable.
